# Different sterilization and disinfection methods used for human tendons – a systematic review using mechanical properties to evaluate tendon allografts

**DOI:** 10.1186/s12891-021-04296-4

**Published:** 2021-04-30

**Authors:** Denes Farago, Blanka Kozma, Rita Maria Kiss

**Affiliations:** 1 Cooperation Research Center for Biomechanics, Faculty of Mechanical Engineering, Budapest University of Technology and Economics, Budapest, Hungary; 2 Department of Mechatronics, Optics and Mechanical Engineering Informatics, Faculty of Mechanical Engineering, Budapest University of Technology and Economics, Budapest, Hungary; 3 Department of Biomedical Engineering, SUNY University at Buffalo, Buffalo, USA

**Keywords:** Tendon, Sterilization, Gamma, Ebeam, Ethylene oxide, SCCO2, Biocleanse, Mechanical properties

## Abstract

**Background:**

It is important to know the biomechanical properties of an allograft. This is because when looking to do a transplant of a tendon, the tendon must have very similar biomechanical properties to the original tendon. To use tendon allografts, it is critical to properly sterilize the tendon before implantation. In past decades, several sterilization procedures have been used. This study aimed to systematically evaluate the existing literature to compare the values of failure load/ultimate strength and Young’s modulus of elasticity of different sterilization methods on commonly used tendon allografts. Five major scientific literature databases (Web of Science, Science Direct, Scopus, PLOS ONE, Hindawi) and additional sources were used.

**Results:**

Studies used had to show a particular sterilization method. Studies were identified to meet the following inclusion criteria: is a controlled laboratory study, gamma irradiation (dose reported), and other sterilization methods. Search for publications dated between 1991 and March 31st, 2020. The database search and additional sources resulted in 284 records. Two hundred thirty records eliminated during the screening for various reasons. The number of articles used in the final synthesis was 54.

**Conclusions:**

Identified sterilization methods (gamma irradiation, ethylene oxid, supercritical carbon dioxide (SCCO2), BioCleanse, Electron Beam) are offered as a catalog of potential methods.

As a result of the broadness of the present research, it provides an overview of sterilization methods and their effect on the mechanical properties (failure load and Young’s modulus of elasticity) of tendons. It does not stand for the state-of-the-art of any single process. Based on a systematic literature review, we recommend freezing and gamma irradiation or electron beam at 14.8–28.5 kGy. These methods are effective at keeping or improving the mechanical properties, while fully sterilizing the inside and the outside of the tendon. Other sterilization method (ethylene oxide, supercritical carbon dioxide (SCCO2), BioCleanse) deteriorated the mechanical properties. These methods are not recommended.

**Supplementary Information:**

The online version contains supplementary material available at 10.1186/s12891-021-04296-4.

## Background

In orthopedic reconstruction, the use of tendon allografts has become more widely accepted in recent years, especially in the anterior cruciate ligament reconstruction. There have been multiple studies conducted which back the use of tendon allografts. The benefits include reduced operation time, reduced donor site morbidity, and unaltered mechanics secondary to harvesting. Additionally, studies conducted on animals and humans have demonstrated that soft tissue allografts are statistically comparable to autografts on an anatomical and biomechanical basis [1–3].

Anterior cruciate ligament (ACL) reconstruction is a common procedure in orthopedic practice [4–6]. A critical decision to be made is the choice of graft. Despite autografts proving capable and displaying good outcomes, graft harvest can cause continual pain at the site of harvest and limit the range of motion. For this reason, a significant increase in allograft use can be seen in the last decade, and despite the higher costs, it remains a feasible choice, specifically in revision cases [4, 7–9]. In young patients, an increased rate of failure of allografts has been recorded. This mostly seems to be caused by the sterilization process, as some studies also reported increased failure rates when comparing fresh-frozen allografts and autografts [1, 6, 10–12]. A couple of studies displayed more inferior results in sterilization compared with fresh-frozen allografts. Septic arthritis after ACL reconstruction has been reported as a risk factor for unsterilized allografts. Also, higher rates of postoperative infections have been reported when allografts were aseptically processed rather than irradiated allografts or autografts [13–15].

A potential complication of using allografts is the risk of disease transmission. Possible diseases include both infections from HIV, Hepatitis C, and bacterial infection from organisms [10, 16, 17]. Whilst the prevalence of the transmission of the disease via allograft tissue is minimal; the potential impact is high [5, 18]. After multiple documented cases of sepsis and infection following ACL reconstruction using allografts, the bacterial transmission of diseases has come to the forefront. These cases were associated with non-irradiated tissue grafts [19–21]. For this reason, an effort has been made to reduce the scarce but possibly disastrous occurrences of bacterial infection by using enhanced sterilization techniques [10, 22–25].

According to standards for tissue banking such as American Association of Tissue Banks (AATB) Standards [26] listed the following for tissue sterilization: radiation (gamma, electron beam and X-ray) and ethylene oxide. Both methods can kill all microorganisms and can be validated according to international standards (ISO documents) and use of biological indicators. WHO and Pharmacoepea accept both sterilization methods for tissues, as these are effective in killing microorganism.

### Aim of the study

The present systematic literature review aims to identify and to categorize existing sterilization methods and their effects on the mechanical properties (failure load/ultimate tensile strength and Young’s modulus of elasticity) of the tendons.

Combining the collected materials should allow us to investigate how different methods can differentiate between participant groups (tendon types) and which methods are more encouraging in research or clinical practice.

## Materials and methods

### Search strategy

#### Identification of materials

This systematic review was executed according to the PRISMA guidelines [27]. Five electronic databases were used (Web of Science, Science Direct, Scopus, PubMed, PloS ONE, and Hindawi) to search for publications dated between 1991 and March 31st, 2020. Key search terms used with Boolean conjunction included: tendon, allograft tendon, tendon sterilization, biomechanical testing, mechanical properties, and synonyms of these terms. The expressions were matched to each database (Table 1).

**Table 1:** Viewpoints and their inclusion criteria and exclusion criteria

Viewpoints	Inclusion criteria	Exclusion criteria
**Tendon irradiation and mechanical test**	Studies that included tendon sterilization and mechanical tests in their experimental processes.	Studies which only included tendon sterilization method without any type of mechanical tests.
**Description of tendon sterilization and mechanical test**	Studies with a detailed explanation of the tendon sterilization and mechanical test and the experimental procedure that was completed.	Studies without detail or incomplete descriptions of the tendon irradiation and mechanical test and the experimental procedures that were completed.
**Assessment of results**	Studies with unbiased results based on measurable parameters.	Studies with biased scoring/assessments of results, not (fully) based on measurable parameters.

For example, a search strategy used for the Science Direct database is as follows. In the Advanced search, the following phrases were added in All fields: (allograft tendon OR tendon) AND (tendon sterilization OR ligament sterilization OR sterilization) AND (biomechanical properties OR mechanical properties OR modulus). The search was refined to journal publications. Publication dates were set from 2008, with the search performed on March 31st, 2020. This search in the Science Direct database yielded 82 records. Key search terms were identified and agreed upon by DF and RMK; electronic search and downloading of results were conducted by DF. Screening, eligibility check of materials, and data extraction were executed by DF, BK, and BS.

#### Screening materials

The associated materials were screened based on title and abstract, removing duplicate entries. The materials of unrelated topics, aims, or completely theoretical work was excluded. Proof of concept articles was not omitted.

#### Eligibility check of materials

Inclusion and exclusion criteria were determined to check for eligibility. Studies had to meet all the inclusion criteria to be included in the final synthesis (Table 1). Studies that either met an exclusion criterion or otherwise failed to meet inclusion criteria were excluded. These criteria were created to provide a quality assessment to a certain extent, i.e., the methods applied had to be well communicated, and the evaluation of the measurement results had to be objective. No additional quality assessment was carried out on the materials included.

#### Data extraction

In compliance with the objective of this research, the final overview of the types of sterilization was to extract relevant information on the assessment of mechanical properties. The data collected from the articles included a) author and date, b) type of tendon, c) type of sterilization, d) sterilization dose, and g) measured and calculated parameters such as failure load/ultimate tensile strength and Young’s modulus of elasticity.

## Results

The database search and additional sources resulted in 284 records (Figure 1, in Supplementary file). One hundred fifty-two records remained after the removal of duplicates and the records with missing or unavailable abstracts. The title and abstract screening eliminated 60 records because of being unfit articles. The 92 items which remained went through a full-text eligibility check. Thirty-eight publications were eliminated from the 92 publications for various reasons, including clinical case reports, publications not written in English, conference abstract, and book chapters. Eight papers were literature review articles related to sterilization methods. The review articles found had an aim and scope different from the present study. The number of articles used in the final synthesis was 54 (*n* = 54).

The following were the reasons for exclusion: several studies applied criteria outside of the scope of our definition of tendon irradiation and mechanical properties, e.g., effects of gamma radiation if the tendon was infected with HIV. If multiple studies described similar tendon irradiation with an equal measurement method and similar instrumentation for assessment, the newest publication was used (30 pieces). Several theoretical articles did not detail a specific measurement setup and were therefore excluded (32 pieces). Some articles used a biased scoring assessment (6 pieces), and a few articles had an unrelated aim of the study, e.g., clinical case (7 pieces).

### Type of sterilization

These studies evaluated the effect of sterilization’s type: non-sterilized and non-frozen tendons (fresh tendons) ([28–31]; Table 2), gamma irradiation ([28, 37–47]; Table 3), Supercritical carbon dioxide (SCCO2) ([28]; Table 4), Ethylene Oxide ([51]; Table 5), BioCleanse ([8, 32–34]; Table 6), Electron Beam (E-beam) ([31, 48, 49, 53, 54]; Table 7), Peracetic acid-ethanol in combination with low-dose gamma irradiation (PE-R) ([35]; Table 8), Hydrogen peroxide ([55]; Table 9) and chlorhexidine ([36]; Table 10). The structure of the tendons may be destroyed by sterilization, so the method used is important. The following subsections detailed these sterilization methods, and the section of conclusion compared the failure load and Young’s modulus of elasticity results found in the literature.

**Table 2:** Average result of failure load and Young’s modulus of elasticity of non-sterilized and non-frozen tendons [8, 28–36]

Type of tendon	Pieces	Failure load (Standard Deviation) [N]	Young’s modulus of elasticity (Standard Deviation) [MPa]	Authors
**Peroneus longus**	14	2091.6 (148.7)	90.3 (11.3)	Aguila et al.
**Peroneus brevis**	20	1485.7 (209.3)	82.4 (19.0)	
**Anterior or posterior tibialis**	15	2427.3 (682.8)	75.4 (30.0)	Baldini et al.
**Patellar tendons**	10	–	25.0 (9.0)	Suhodolcan et al.
**Human flexor digitorum superficialis**	10	402.5 (38.4)	400.5 (58.5)	Wei et al.
**Patellar tendon**	14	-	158.0 (49)	Bechtold et al.
**Achilles**	10	3032 (677)	292.04 (123.15)	Conrad et al.
**Tibialis anterior**	12	1665 (291.3)	19.9 (4.7)	Schimizzi et al.
**Tibialis tendon**	10	606.73 (283.52)	213.13 (98.86)	Elenes et al.
**Bone – patellar tendon – bone**	10	1741 (304)	–	Hoburg et al. Hoburg et al.
**Human flexor tendons**	–	360.01 (88.17)	221.55 (73.06)	Zhou et al.
**Patellar tendon**	8	1878 (613)	–	Sobel et al.

**Table 3:** Average result of failure load and Young’s modulus of elasticity of gamma-irradiated tendons [8, 29, 33, 34, 37–50]

Type of tendon	Irradiation dose	Pieces	Failure load(Standard Deviation) [N]	Young’s modulus of elasticity (Standard Deviation) [MPa]	Authors
**Peroneus longus**	1.5–2.5 Mrad	14	2122.8 (380.0)	94.8 (21.0)	Aguila et al.
**Peroneus brevis**	1.5–2.5 Mrad	20	1318.4 (296.9)	72.5 (16.6)	
**Patellar tendon**	18.3–21.8 kGy	9	2410 (1100)	88.11 (26.9)	Balsly et al.
**Patellar tendon**	24.0–28.5 kGy	9	2410 (930)	72.44 (21.30)	
**Anterior tibialis**	18.3–21.8 kGy	10	2890 (720)	328.47 (37.12)	
**Anterior tibialis**	24.0–28.5 kGy	10	2420 (330)	309.66 (56.67)	
**Semitendinosus**	18.3–21.8 kGy	8	1010 (360)	369.08 (122.47)	
**Semitendinosus**	24.0–28.5 kGy	10	1230 (380)	410.08 (98.86)	
**Fascia lata**	18.3–21.8 kGy	10	460 (140)	366.27 (87.38)	
**Fascia lata**	24.0–28.5 kGy	10	420 (190)	238.51 (113.43)	
**Anterior or posterior tibialis (young)**	1.48–1.80 Mrad	10	3062 (699)	–	Greaves et al.
**Anterior or posterior tibialis (middle)**	1.48–1.80 Mrad	13	2729 (995)	–	
**Anterior or posterior tibialis (old)**	1.48–1.80 Mrad	10	3004 (603)	–	
**Flexor digitorum superficialis**	25 kGy	10	335.96 (28.32)	357.72 (43.97)	Ren et al.
**Flexor digitorum superficialis**	25 kGy (freeze thaw)	10	287.41 (23.20)	346.95 (69.09)	
**Achilles**	18–24 kGy	10	3572.54 (393.57)	181.7 (24.05)	Hangody et al.
**Achilles**	38–46 kGy	10	392.01 (180.91)	134.75 (15.07)	
**Quadriceps**	18–24 kGy	10	3184.32 (101.62)	302.96 (45.71)	
**Quadriceps**	38–46 kGy	10	3464.35 (462.45)	192.28 (51.88)	
**Semitendinosus + gracilis**	18–24 kGy	10	2310.32 (561.2)	248.93 (14.43)	
**Semitendinosus + gracilis**	38–46 kGy	10	2271.96 (651.12)	213.88 (20.28)	
**Tibialis anterior**	18–24 kGy	10	3107.76 (606.41)	327.93 (44.31)	
**Tibialis anterior**	38–46 kGy	10	2678.96 (181.45)	375.15 (67.84)	
**Peroneus longus**	18–24 kGy	10	2631.81 (297.94)	284.84 (16.03)	
**Peroneus longus**	38–46 kGy	10	2291.71 (523.76)	333.11 (79.11)	
**Patellar tendon**	4 Mrad	16	1884 (330)	–	Rasmussen et al.
**Achilles**	1.5–2.5 Mrad	10	1972 (558)	129.48 (53.22)	Conrad et al.
**Tibialis anterior**	20–26 kGy	12	1671.9 (290.2)	22.6 (5.9)	Schimizzi et al.
**Tibialis tendon**	17.1–21 kGy	10	597.09 (280.32)	179.02 (73.13)	Elenes et al.
**Bone – patellar tendon – bone**	25 kGy	10	1009 (400)	–	Hoburg et al. Hoburg et al.
**Bone – patellar tendon – bone**	34 kGy	10	1073 (617)	–

**Table 4:** Average result of failure load and Young’s modulus of elasticity of sterilized tendons with supercritical carbon dioxide [28]

Type of tendon	Pieces	Failure load(Standard Deviation) [N]	Young’s modulus of elasticity (Standard Deviation) [MPa]	Authors
**Anterior or posterior tibialis**	11	2450.3 (576.8)	91.9 (30.2)	Baldini et al.

**Table 5:** Average result of failure load and Young’s modulus of elasticity of sterilized tendons with ethylene oxide [51]

Type of tendon	Pieces	Failure load(Standard Deviation) [N]	Young’s modulus of elasticity (Standard Deviation) [MPa]	Authors
**Patellar tendon**	14	–	143.0 (67.0)	Bechtold et al.

**Table 6:** Average result of failure load and Young’s modulus of elasticity of sterilized tendons with BioCleanse [8, 32–34, 52]

Type of tendon	Pieces	Failure load(Standard Deviation) [N]	Young’s modulus of elasticity (Standard Deviation) [MPa]	Authors
**Achilles**	10	2472 (701)	154.06 (104.54)	Conrad et al.
**Tibialis anterior**	12	1559 (176)	484.2 (99.1)	Colaco et al.
**Tibialis anterior**	12	1651.6 (377.4)	19.7 (5.4)	Schimizzi et al.

**Table 7:** Average result of failure load and Young’s modulus of elasticity of sterilized tendons with Electron Beam [31, 48, 49, 53, 54]

Type of tendon	Irradiation dose	Pieces	Failure load(Standard Deviation) [N]	Young’s modulus of elasticity (Standard Deviation) [MPa]	Authors
**Tibialis tendon**	9.2–12.2 kGy	10	876.38 (310.73)	206.71 (88.87)	Elenes et al.
**Tibialis tendon**	17.1–21.0 kGy	10	660.24 (312.12)	152.64 (75.10)	
**Human flexor digitorum superficialis**	50 kGy	10	282.3 (53.0)	291.6 (50.8)	Wei et al.
**Human flexor digitorum superficialis**	50 kGy, fractionation	10	360.9 (49.3)	354.3 (49.1)	
**Human flexor digitorum superficialis**	50 kGy, fractionation + ascorbate	10	390,6 (42.1)	390.4 (50.6)	
**Bone – patellar tendon – bone**	34 kGy	11	1024 (204)	–	Hoburg et al.
**Bone – patellar tendon – bone**	34 kGy, fractionated	11	1327 (305)	–	
**Bone – patellar tendon – bone**	25 kGy	10	1177 (512)	–	Hoburg et al.
**Bone – patellar tendon – bone**	34 kGy	10	1139 (445)	–

**Table 8:** Average result of failure load and Young’s modulus of elasticity of sterilized tendons with PE-R [35]

Type of tendon	Pieces	Failure load(Standard Deviation) [N]	Young’s modulus of elasticity (Standard Deviation) [MPa]	Authors
**Human flexor tendons**	–	306.96 (61.52)	235.78 (96.13)	Zhou et al.

**Table 9:** Average result of failure load and Young’s modulus of elasticity of sterilized tendons with Hydrogen peroxide [55]

Type of tendon	Pieces	Ultimate tensile strength (Standard Deviation) [MPa]	Young’s modulus of elasticity (Standard Deviation) [MPa]	Authors
Tibialis posterior	5	2366 (447)	–	**Gardner et al.**
Tibialis anterior	2	2308 (806)	–	
Extensor digitorum longus	6	1574 (116)	–	
Extensor halluces logus	5	588 (16)	–	
Flexor digitorum longus	1	1087	–	
Flexor halluces logus	3	1210 (155)	–

**Table 10:** Average result of failure load and Young’s modulus of elasticity of sterilized tendons with Chlorhexidine [36]

Type of tendon	Pieces	Failure load(Standard Deviation [N]	Young modulus of elasticity [MPa]	Authors
Patellar tendon	8	2219 (808.1)	–	Sobel et al.

### Non-sterilized tendon (fresh/control tendon)

Four articles mention the fresh grafts, which did not receive any sterilization ([8, 28–36]; Table 2). After they were procured from human cadavers, they were placed in a saline solution and cooled until the measurement. In comparative studies, non-sterilized tendons are measured as a standard to be able to tell to what extent the sterilization method and dose change its properties. Baldini et al. [28] analyzed three groups of anterior and posterior tibialis tendons. The results showed that the failure load and Young’s modulus of elasticity of the fresh group are weaker than the gamma-irradiated and SCCO2 sterilized groups ([28]; Table 2). Aguila et al. [29] found that no differences were between the results of the failure load and Young’s modulus of elasticity of the non-sterilized and the gamma-irradiated groups in peroneus brevis and peroneus longus ([29], Table 2). Suhodolcan et al. [30] also compared three groups (fresh, frozen, and cryopreserved) of the patellar tendon; they compared the results of the fresh group to the frozen and the cryopreserved ([30], Table 2). Wei et al. [31] found that the mechanical properties (failure load and Young’s modulus of elasticity) of the fresh group were higher than in the gamma-irradiated group ([31]; Table 2). From the above results, it can be stated that the values of the failure load and Young’s modulus of elasticity of sterilized tendons either did not change or decrease as a result of gamma and gamma-irradiated ([8, 28–36]; Table 2).

#### Gamma irradiation

Gamma radiation is one of the most common sterilization methods. This sterilization method is used by ten articles ([8, 29, 33, 34, 37–50]; Table 3). During radiation, two doses are distinguished, the low dose (bacterial dose) of 15–26 kGy and the high dose (viricidal dose) of 30–45 kGy [44, 47]. The dosage unit is Mrad or kGy, 1 Mrad = 10 kGy.

The temperature can influence the impact of irradiation. This is important for interpreting results that is why we should point out this further nuance. Typically, it is cooled to between − 20 °C and − 80 °C prior to sterilization and the tendon temperature is kept down by dry ice using gamma radiation. However, one article [44] compares the properties of tendons irradiated at room temperature (20 °C) and those frozen in freezing (− 72 °C), which states that weaker properties are obtained for fresh tendons irradiated at room temperature. The articles were split into two groups; in the first group, multiple doses were used ([37, 40, 46]; Table 3). In the second group, only one dose was used ([29, 38, 39, 41–43, 45]; Table 3). Balsly et al. [37] used two irradiation doses, where the absorbed dose is 18.3–21.8 kGy and 24–28.5 kGy. Four tendons (patellar, anterior tibialis, semitendinosus, and fascia lata) were examined. Before the irradiation the tendons were deepfreeze to − 80 °C and during the sterilization were on dry ice. Based on the results, it could not be declared that the increasing dose value diminished or increased the failure load and Young’s modulus of elasticity of the tendons [37]. Gut et al. [40] used four doses (25, 35, 50, 100 kGy) for the patellar tendon. Before the irradiation the tendons were deepfreeze to − 70 °C and during the sterilization were on dry ice. The failure load of the irradiated tendons deteriorated relative to the control group. With an increase in dose, the mechanical properties of the tendon improved to 35 kGy, and after that, they deteriorated [40]. In the article by Hangody et al. [46], the target doses were 21 and 42 kGy, and five different types of tendons were tested. Before the irradiation the tendons were deepfreeze to − 72 °C and during the sterilization were on dry ice. In the case of the 21 kGy dose, the failure load and Young’s modulus of elasticity of tendons improved versus the frozen group. In the case of the 42 kGy dose, the mechanical properties of some grafts improved, and others deteriorated [46].

In the second group, the effect of gamma irradiation was examined compared to the control group. The values between 14.6–40 kGy radiation dose were examined. In two cases [39, 42], the gamma radiation improved the failure load and Young’s modulus of elasticity of the tendons. Weber et al. used a 15–25 kGy irradiation dose for the Achilles tendon [42]. Curran et al. [39] used a 20 kGy irradiation dose for the patellar tendon. Before the irradiation the tendons were deepfreeze to − 20 °C and during the sterilization were on dry ice. The gamma irradiation caused a deterioration in failure load, and Young’s modulus of elasticity [29, 41, 43, 45] Aguila et al. [29] used 15–25 kGy for peroneus longus and peroneus brevis, Deyne and Haut [41] used 20 kGy for patellar tendons, Ren et al. [43] used 25 kGy for flexor digitorum superficialis, and Rasmussen et al. [45] used 40 kGy for the patellar tendon. Greaves et al. [38] compared three age groups (“young”, “middle”, “old”). Deterioration can be observed in the “middle” age group and the “old” age group. There are values of failure load, and Young’s modulus of elasticity, no noticeable change in the “young” age group [38].

#### Supercritical carbon dioxide (SCCO2)

Supercritical carbon dioxide is an alternative sterilization method ([28], Table 4). We found one relevant article on this method [28], but the article compares several sterilization methods. The allografts were secured in their casing and placed in a chamber with heated and pressurized CO_2,_ which forms a solvent that sterilizes the allograft. Baldini et al. [28] compared the SCCO2 method with gamma irradiation in anterior and posterior tibialis. Based on the measurements, the SCCO2 treated grafts show higher values of failure load and Young’s modulus of elasticity than the gamma irradiation group ([28], Table 4).

#### Ethylene oxide

We found one relevant article on this method [51]. Bechtold et al. [51] used ethylene oxide for sterilization ([51], Table 5), and the study presented two types of freezing as a control group. The grafts are assigned to freeze-drying and sterilization. The tendon is exposed to 12% ethylene oxide gas at 32 °C for 15 h, with a relative humidity of approximately 80% before being freeze-dried. This alternative procedure does not improve the failure load and Young’s modulus of elasticity of the tendons ([51], Table 5).

#### BioCleanse

Following the BioCleanse protocol, all tendons were closed in a chamber and were exposed to a solvent at differing pressures and vacuum cycles ([8, 32–34, 52]; Table 6). The treatment group was exposed to the chemical solutions for twice as long as the standard time of exposure. This was designed to portray the worst outcome for degrading the structural and mechanical characteristics of the donor material [52]. Based on the results, the failure load and Young’s modulus of elasticity showed weaker results than gamma irradiation group ([8, 32–34, 52]; Table 6).

#### Electron beam (E-beam)

The sterilization method did not differ much from the traditional gamma irradiation method ([31, 48, 49, 53, 54]; Table 7). The tendon grafts were encased in a specially created Styrofoam box, packed with dry ice to preserve the approximate temperature of − 40 °C to − 50 °C throughout the complete irradiation process. Stronger mechanical properties are achieved by the E-beam method ([31, 48, 49, 53, 54]; Table 7) compared to the method with gamma irradiation, but there were no differences.

#### Peracetic acid-ethanol in combination with low-dose gamma irradiation (PE-R)

This hybrid method consists of chemical sterilization and gamma radiation ([35]; Table 8). We found one relevant article on this method [35], but the article compares several sterilization methods. The PAA/ethanol (PE) solution was prepared using distilled water and absolute ethanol. It consisted of 0.2% PAA and 24% ethanol. Tendons were submerged in the PE solution for 30 min and later washed with a saline solution until the concentration of PAA was lower than 1 ppm. The refined human flexor tendons were then closed and subjected to gamma irradiation at an average dose of 15 kGy. The sterilized muscles were stowed at − 80 °C [35], and they showed stronger mechanical results than the control group. This hybrid procedure has not been compared with other sterilization methods ([35]; Table 8).

#### Hydrogen peroxide

We found only one relevant article on this method [55], but the article summarized the effect of hydrogen peroxide on several tendons. This chemical process requires that 3% hydrogen peroxide be added to the solution ([55]; Table 9). The tissues should be left in the solution for 5 min in room temperature and the tendons were stored at − 80 °C. This is the time needed to kill the bacteria. Based on the results of the study, there is no change in the mechanical properties of treated and untreated tendons. Gardner et al. did not compare this method with other sterilization methods. This is sufficient to kill bacteria but not enough to kill viruses ([55]; Table 9).

#### Chlorhexidine

In this chemical process, the experimental group is immersed in a 4% chlorhexidine gluconate solution for 30 min, while the control group was kept moist in saline gauze. The results of patellar tendons were not compared with other sterilization procedures [36]. The experimental group had stronger mechanical properties than the control group, this property does not result in tissue alterations that adversely affect its clinical application ([36]; Table 10). We found one relevant article on this method, but the article compares several sterilization methods.

## Discussion

The two factors that can affect the mechanical properties of a tendon are the type of tendon and the sterilization methods. Tendon grafts show to be promising for transplants. It is important to sterilize the tendons to ensure no bacteria is transmitted that could cause infections. We reviewed peroneus longus, peroneus brevis, tibialis anterior, tibialis posterior, patellar tendon, human flexor digitorum superficial, Achilles, bone-patellar tendon-bone, semitendinosus, semitendinosus + gracilis, fascia lata types of tendon and gamma irradiation, SCCO2, ethylene oxide, BioCleanse, E-Beam, PE-R, hydrogen peroxide, chlorhexidine sterilization methods.

Gamma radiation is the most common method of sterilization (Table 3). The 14.8–28.5 kGy (1.48–2.85 Mrad) radiation proved best because this radiation dose killed bacteria, according to Document ISO 11137 [56], 15 kGy can sterilize tendon if bioburden is less than 1,5 cfu/item and 25 kGy can sterilize tendon if bioburden not more than 1000 cfu/item. Many tissue banks have developed clean rooms whereby the processing of tendon is carried out under aseptic environment. The tissues have very low microorganism count or bioburden and mostly no bacterial count at all. As radiation doses can be selected and validated based bioburden in the tissue, there is a tendency that many tissue banks would want to irradiate soft tissues at doses lower than 25 kGy (the most commonly used radiation dose) therefore will avoid effects of radiation on mechanical properties. It also kept the failure load (e.g., failure load of Achilles sterilized at18–24 kGy is 3572.54 N vs. failure load of Achilles sterilized at 38–46 kGy is 392.01 N, tibialis anterior sterilized at 18–24 kGy: 3107.76 N, failure load of tibialis anterior sterilized at 38–46 kGy: 2678.96 N [45, 46]. Comparing with section 3.1.1 (non-sterilized tendon), the results showed that failure load improved (Achilles: 3032 N, tibialis anterior: 2427.3 N). It can be observed that the failure load’s tendency of quadriceps was different (18–24 kGy: 3184.32 N, 38–46 kGy: 3464.35 N). At 38–46 kGy (3.8–4.6 Mrad), the radiation killed viruses as well, but the biomechanical properties deteriorate (Tables 2 and 3) [29, 37–47];.

The effectiveness of sterilization depends on the temperature and the dose. Samples should be cooled to at least − 70 °C prior to sterilization, and using dry ice or liquid nitrogen, samples should also be cooled during gamma irradiation, as heat is generated during the procedure, which can cause tendons to melt and decompose, leading to tissue damage. At high doses> 25 kGy, tissue damage and poorer mechanical properties are also observed, therefore virucidal sterilization is not recommended. Viruses can be detected during screening tests and infected samples can be excluded from further use. The supercritical carbon dioxide (SCCO2) sterilization method has been discussed only by Baldini et al. [28]. Baldini et al. discussed anterior or posterior tibial tendon treated with SCCO2. Based on the results, there was a negligible improvement in the failure load and Young’s modulus of elasticity of the tendons (non-sterilized: 2427.3 N, 75.4 MPa vs. SCCO2: 2450.3 N, 91.9 MPa) ([28]; Tables 2 and 3). The origin of samples and measurement methods are different; the result could not be compared to the results of different sterilization methods. Therefore, we cannot accurately conclude.

The Ethylene oxide procedure does not yield higher mechanical properties than other methods ([51]; Table 5). Tendons sterilized with this method generate connective tissue growth. During this tissue growth, cancerous cells can develop. Bechtold et al. [48] examined the patellar tendon and its Young’s modulus of elasticity. After sterilization, Young’s modulus of the tendon deteriorated slightly (non-sterilized: 158.0 MPa, sterilized: 143.0 MPa). This sterilization method is also not comparable to other sterilization methods, but the method impaired Young’s modulus of the patellar tendon [51].

The BioCleanse wash poses the same issues as the hydrogen peroxide and chlorhexidine method ([32–34, 52]; Table 6). Since this is a wash, only the outside of the tendon is sterilized. Dangerous bacteria might still be found in the tendon. The BioCleanse solution is not as effective as gamma irradiation. The sterilization method is addressed in three studies (Table 6). Conrad et al. [8] examined the failure load and Young’s modulus of the Achilles after different sterilization. BioCleanse and gamma irradiation sterilization methods are investigated, and compared the result of mechanical properties of non-sterilized tendon. In the case of failure load, BioCleanse shows lower results than non-sterilized, but even weaker are the results of the gamma irradiation sterilization method (non-sterilized: 3032 N, BioCleanse: 2472 N, gamma irradiation: 1972 N). The values of the modulus of elasticity showed a similar tendency (292.04–154.06-129.48 MPa) [8]. Colaco et al. [32] and Schimizzi et al. [33], the tibialis anterior was examined. Colaco et al. [32] compared different BioCleanse mixtures. However, no comparison was made with other sterilization methods. Schimizzi et al. [33] analyzed the effect of sterilization by BioClense and gamma irradiation on the mechanical properties of tibialis anterior. The results were compared to mechanical properties of non sterilized tendon.. Based on the results, BioCleanse did not achieve lower results (failure load and Young’s modulus) compared to the other two groups (non-sterilized: 1665 N and 19.9 MPa, gamma-irradiated: 1671.9 N and 22.6 MPa, BioCleanse: 1651.6 N and 19.7 MPa) [33].

The effect of sterilization by Electron Beam (E-beam) was analyzed in three articles ([31, 48, 53], Table 7). Elenes et al. [48] compared failure load of the tibialis anterior with non-sterilized, gamma irradiation, and E-beam sterilization methods. It uses two types of E-beam doses, one low (9.2–12.2 kGy) and one high (17.1–21.0 kGy). Based on the maximum load results [53], the low-dose E-beam performed significantly higher compared to the other three categories (non-sterilized: 606.73 N, E-beam low: 876.38 N, E-beam high: 660.24 N, gamma: 597.09 N). In comparison to the values of modulus of elasticity, it can be observed that the values decrease for all three sterilization methods compared to non-sterilized ones. Of the three sterilization methods, the smallest deterioration is shown by E-beam low (non-sterilized: 213.13 MPa, E-beam low: 206.71 MPa, E-beam high: 152.64 MPa, gamma: 179.02 MPa) [48]. Wei et al. [31] compared three types of E-beam doses to the non-sterilized group. Based on the results, all three doses of E-beam performed lower than the non-sterilized human flexor digitorum superficialis (fresh: 402.5 N and 400.5 MPa, 50 kGy: 282.3 N and 291.6 MPa, 50 kGy (Fr.): 360.9 N and 354.3 MPa, 50 kGy (Fr. + As.): 390.6 N and 390.4 MPa) [31]. Hoburg et al. [49] examined the E-beam in the most recent of his studies. Bone-patellar tendon-bone treated with two doses of E-beam radiation (25 and 34 kGy) was compared with two different doses of gamma radiation (25 and 34 kGy) and non-sterilized. Based on the results, all four sterilizations significantly decreased the maximum force values; however, the values of E-beam are stronger than the values of the same dose of gamma radiation (non-sterilized: 1741 N, gamma 25 kGy: 1009 N, E-beam 25 kGy: 1177 N, gamma 34 kGy: 1073 N, E-beam 34 kGy: 1139 N) [49]. Zhou et al. [35] examined human flexor tendon sterilized with Peracetic acid - ethanol in combination with low-dose gamma irradiation (PE-R). The results showed a lower failure load compared to the non-sterilized tendon (non-sterilized: 360.01 N and 221.55 MPa, PE-R: 306.96 N, and 235.78 MPa). PE-R is a hybrid method consisting of immersing the tendon in a chemical solution and then using gamma irradiation. This method yielded a lower failure load than the Electron beam sterilization and gamma sterilization [35].

The hydrogen peroxide method is just a wash; the inside of the tendon is not properly sterilized, which can lead to infections. Gardner et al. [55] examined the effect of hydrogen peroxide on several different types of tendons. The ultimate tensile strength was not compared to other sterilization methods or non-sterilized tendons (Table 9). Comparing the results of the ultimate tensile strength of previous studies with the results, hydrogen peroxide is not a suitable method for sterilizing tendons [55].

The Chlorhexidine chemical solution is also a wash, the same as the hydrogen peroxide method [36]. While it does pose higher results of failure load for the tendon, the inside of the tendon is not properly sterilized. According to the method of Sobel et al. [36] showed that the value of the failure load increases as a result of sterilization (non-sterilized: 1876 N, Chlorhexidine: 2219 N) (Table 10).

Based on a systematic literature review, it can be established that the most common method of sterilization is gamma radiation. However, after comparing the different literature, it is established that mechanical properties are improved, compared to non-sterilized tendons, with the E-beam sterilization method. The method is not widespread, due to the lack of laboratories and instruments suitable for conducting this treatment. The procedure using Chlorhexidine has yielded similar results to the E-beam. Only a few studies are available on the subject, so further research is needed. Gamma and electron sterilization service is offered by irradiation facilities and no lab-scale instrument for electron beam is available. Even self-shielded Gammacell with small irradiation compartment is extremely expensive and very costly to maintain and need nuclear personnel to validate. The existing practice is tissue banks will send packages of tissues to certified gamma and electron beam facilities that offer sterilization service.

### Limitation

This review analyzed and classified multiple existing sterilization methods used in determining the biomechanical properties of tendons. Since this review looked at different techniques, a meta-analysis could not be performed. Limitations can also surface because only three major scientific databases were utilized in the search for papers. Because of the full range of this research, it only provides an overview of multiple sterilization processes. This review is not meant to be exhaustive but to provide manuscript limitation examples. It does not discuss the advancement of any single process. Two studies analyzed the effect of different sterilization methods on the mechanical properties of same tendon [8, 33]. The most research The results of different studies for the same tendon cannot be numerically compared because sterilization method (chemical, radiation), storage and handling are different. In the future, the effect of different sterilization method should be analyzed on the mechanical properties on the same tendon.

## Conclusion

The purpose of this literature review was to systematically evaluate existing literature to compare the biomechanical effects of different sterilization methods on commonly used tendon allografts. The mechanical properties of tendons that were determined included failure load and Young’s modulus of elasticity.

Based on a systematic literature review, we recommend freezing and gamma irradiation or electron beam at 14.8–28.5 kGy. This is especially useful for new bone bankers who prefer to supply non-processed frozen bones that are sterilized by radiation. These methods are effective at keeping or improving the mechanical properties, while fully sterilizing the inside and the outside of the tendon. Other sterilization method (ethylene oxide, supercritical carbon dioxide (SCCO2), BioCleanse) deteriorated the mechanical properties. These methods are not recommended.

## Supplementary Information

**Additional file1: Figure S1.** PRISMA flow diagram of the systemic review process.

## Data Availability

The data that support the findings of this study are available from authors of not open access journals but restrictions apply to the availability of these data, which were used under license for the current study, and so are not publicly available. Data are however available from Denes Farago upon reasonable request and with permission of authors of not open access journals. All data generated or analysed during this study are included in this published review.

## Abbreviations

ACL: Anterior cruciate ligament
SCCO2: supercritical carbon dioxide
E-beam: Electron Beam
PE-R: Peracetic acid-ethanol in combination with low-dose gamma irradiation
